# Editorial: Personality interactions in the workplace: light and dark sides of human behavior

**DOI:** 10.3389/fpsyg.2026.2012061

**Published:** 2026-10-20

**Authors:** Manuel Galán, David Pineda, Ben-Roy Do

**Affiliations:** 1 Department of Psychology, Catholic University San Antonio of Murcia, Murcia, Spain; 2 Department of Health Psychology, Miguel Hernández University of Elche, Elche, Spain; 3 Department of Psychology, Fu Jen Catholic University, New Taipei, Taiwan

**Keywords:** Dark Tetrad, dyadic processes, interpersonal interactions, social exchange theory, workplace relationships

Personality traits have long been recognized as important predictors of individual performance, with the Five-Factor Model (FFM) providing a widely adopted framework for understanding these relationships (Barrick and Mount, 1991). Contemporary organizational researchers increasingly adopt interactionist and trait activation perspectives (Tett and Burnett, 2003), emphasizing that the expression of personality traits depends on situational cues and contextual conditions.

Consistent with this perspective, scholarly attention has increasingly expanded beyond traditionally studied positive personality characteristics to encompass both the “light” and “dark” sides of personality and human behavior (Kaufman et al., 2019). While prosocial attributes may promote constructive and citizenship behaviors, aversive personality traits can produce more complex and context-dependent outcomes. In particular, the Dark Tetrad—comprising narcissism, Machiavellianism, psychopathy, and sadism—has attracted growing attention in organizational research because these traits have been associated with both adaptive and maladaptive workplace outcomes (Thibault and Kelloway, 2020).

This Research Topic, “*Personality interactions in the workplace: light and dark sides of human behavior*,” was conceived to advance this dialogue. Bringing together 10 contributing articles comprising quantitative and qualitative investigations across diverse cultural and organizational contexts, as well as a systematic meta-analysis, this Research Topic sheds light on the complex mechanisms in which personality relates to workplace behaviors, employee wellbeing, and organizational issues.

Drawing on social exchange theory, Wu et al. investigate the ripple effects of Machiavellian leadership on subordinate happiness at work. Their findings reveal that leaders high in Machiavellianism may trigger psychological contract breach, which diminishes employees' happiness at work, while high-quality coworker exchange can buffer the negative consequences of Machiavellian leadership. Complementing this focus on subordinates, Li C. et al. adopt an observer's perspective to analyze exploitative leadership, grounded in Social Information Processing Theory. Their study shows that observing a leader exploit colleagues heightens observers' own feelings of job insecurity through psychological contract breach, but a high-quality leader–member exchange (LMX) relationship can reduce individuals' susceptibility to exploitative leaders.

Other contributions examine the implications of dark personality traits for employee wellbeing. Using data from a large national survey in China, Wang et al. examine the dark personality facet of narcissistic rivalry among working adults. Their investigation uncovers a sequential mediation mechanism linking narcissistic rivalry to heightened occupational burnout via elevated perceived stress and deteriorated sleep health. In the pursuit of employee-organization fit, Wnuk observes that employees with elevated Dark Triad traits actually report greater stress and emotional exhaustion when working in a caring ethical climate. This counterintuitive finding suggests the need to match ethical preferences between potential employees and the organization during recruitment.

Addressing unethical behavior based on theory of mind, Albejaidi and Mughal investigate the Dark Triad with the Fraud Pentagon framework among future accounting professionals. Their work elucidates how traits of narcissism, Machiavellianism, and psychopathy activate cognitive states of opportunity perception, rationalization, capability, and intention formation that may predispose individuals toward unethical decision-making.

Turning to constructive workplace behavior and wellbeing, Çukurovali et al. draw on conservation of resources and job demands-resources theories to study proactive personality among insurance sector personnel. Their empirical findings establish that individuals with proactive dispositions engage significantly more in innovative work behavior, which in turn elevates overall job performance, validating personal initiative as a core catalyst for workplace innovation. Grounded in self-determination theory, organizational support theory, and the stimulus–organism–response framework, Abdou and Albohnayh explore autonomy-supportive leadership in the high-stress hospitality industry. Their investigation reveals that autonomy-supportive supervisory styles may satisfy fundamental psychological needs of autonomy, competence, and relatedness and promote lasting employee psychological wellbeing, with perceived organizational support playing a moderating role. Broadening this perspective to ethical factors, in a rigorous systematic review and meta-analysis, Li Z. et al. synthesize the broad umbrella of “ethics” into three distinct conceptual boundaries: individual moral traits and cognitions, interpersonal and leadership dynamics, and macro-level organizational contexts. Relationships between ethical responsibility and organizational citizenship behavior are found to be mediated by ethical leadership, ethical climate, and moral identity.

Relationships among peers also feature in the Research Topic, encompassing both supportive and harmful dynamics. Based on social exchange theory, Zhang et al. examine the psychological mechanisms underpinning the temporal continuity of organizational citizenship helping behavior, perceived team-member exchange, and perceived coworker support. They demonstrate when and how performing good deeds replenishes—or depletes—the actor's willingness to sustain future helping. Finally, venturing into transnational educational settings, Sun et al. utilize qualitative and network perspectives to dissect “moralized reputational governance” and relational aggression within co-national peer networks. Their analysis unpacks how interpersonal surveillance, gossip, and ostracism operate as subtle coercive mechanisms, reflecting covert, relational dark-side behaviors within tight-knit institutional groups.

Taken together, the 10 contributions in this Research Topic show that in organizational and educational settings, behaviors cannot be understood simply in terms of individual traits or “good” and “bad” actors. Across the Research Topic, leadership practices, social exchange relationships, ethical climates, and peer dynamics emerge as recurring elements in understanding harmful and constructive behavior. Together, these contributions highlight the value of studying individual characteristics alongside the interpersonal and institutional contexts in which they are expressed. We hope this Research Topic encourages more longitudinal, dyadic, and network-based research while helping organizational leaders and scholars better understand hidden toxicity, promote ethical behavior, and build healthier and more positive workplaces.
